# HAX1-Overexpression Augments Cardioprotective Efficacy of Stem Cell-Based Therapy Through Mediating Hippo-Yap Signaling

**DOI:** 10.1007/s12015-024-10729-z

**Published:** 2024-05-07

**Authors:** Wen-Feng Cai, Lin Jiang, Jialiang Liang, Suchandrima Dutta, Wei Huang, Xingyu He, Zhichao Wu, Christian Paul, Xiang Gao, Meifeng Xu, Onur Kanisicak, Junmeng Zheng, Yigang Wang

**Affiliations:** 1 Department of Pathology and Laboratory Medicine, College of Medicine, University of Cincinnati, 231 Albert Sabin Way, Cincinnati, OH 45267-0529 USA; 2 Department of Internal Medicine, College of Medicine, University of Cincinnati, Cincinnati, OH 45267-0529 USA; 3 Department of Cardiovascular Surgery, Sun Yat-Sen Memorial Hospital, Sun Yat-Sen University, No.107 Yanjiang West Road, Guangzhou, 510120 Guangdong China

**Keywords:** HAX1, Mst1, Sca1-posistive cardiac stromal cells, Proliferation, Apoptosis, Angiogenesis, Myocardial infarction

## Abstract

Although stem/progenitor cell therapy shows potential for myocardial infarction repair, enhancing the therapeutic efficacy could be achieved through additional genetic modifications. HCLS1-associated protein X-1 (HAX1) has been identified as a versatile modulator responsible for cardio-protective signaling, while its role in regulating stem cell survival and functionality remains unknown. In this study, we investigated whether HAX1 can augment the protective potential of Sca1^+^ cardiac stromal cells (CSCs) for myocardial injury. The overexpression of HAX1 significantly increased cell proliferation and conferred enhanced resistance to hypoxia-induced cell death in CSCs. Mechanistically, HAX1 can interact with Mst1 (a prominent conductor of Hippo signal transduction) and inhibit its kinase activity for protein phosphorylation. This inhibition led to enhanced nuclear translocation of Yes-associated protein (YAP) and activation of downstream therapeutic-related genes. Notably, HAX1 overexpression significantly increased the pro-angiogenic potential of CSCs, as demonstrated by elevated expression of vascular endothelial growth factors. Importantly, implantation of HAX1-overexpressing CSCs promoted neovascularization, protected against functional deterioration, and ameliorated cardiac fibrosis in ischemic mouse hearts. In conclusion, HAX1 emerges as a valuable and efficient inducer for enhancing the effectiveness of cardiac stem or progenitor cell therapeutics.

**Graphical Abstract:**

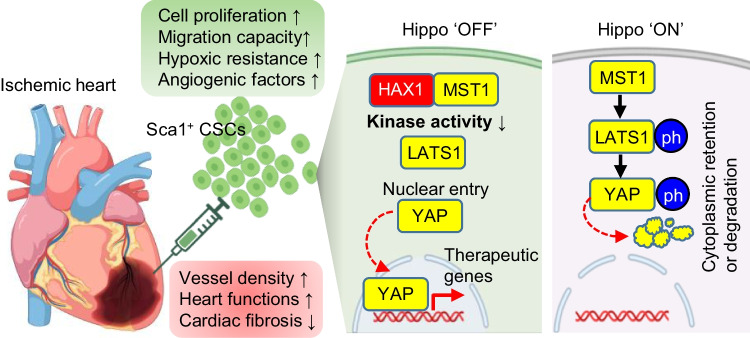

**Supplementary Information:**

The online version contains supplementary material available at 10.1007/s12015-024-10729-z.

## Introduction

HAX1 was originally identified as an interactive component of HS1 (a substrate of Src family tyrosine kinase) that contributes to the maintenance of clonal homeostasis in lymphoid cells [50]. Numerous studies have demonstrated that HAX1 is a multifunctional regulator that participates in several important biological processes including cell proliferation, apoptosis, cell migration, and inflammatory responses. Such a variety of performances are completed by HAX1 through interactions with other cellular signaling pathways [52].

Distinct pro-survival molecular mechanisms of HAX1 have been elucidated (both in vitro and in vivo) in cancer cells, neurons, immune cells, and cardiomyocytes*.* Mechanistically, HAX1 can not only directly inhibit the activation of caspases-3 and -9, the prominent endoproteases responsible for the deliberate disassembly of the cell into apoptotic bodies [17, 20, 26], but also stabilize the intracellular apoptotic constraints, such as XIAP and cIAP, during this programmed death process [7, 20]. However, HAX1 contains a PEST (Polypeptide sequence enriched in proline (**P**), glutamic acid (**E**), serine (**S**), and threonine (**T**)) motif and serves as a proteasome ubiquitin-binding domain, suggesting that this biomolecule may be subjected to rapid destruction in response to stress stimuli [15, 27]. In the heart, HAX1 expression decreased in response to ischemia/reperfusion (I/R) injury, and this reduction consequently led to enhanced cardiomyocyte apoptosis and compromised heart performance [23]. Evidence from the loss-of-function experiments has also demonstrated that endoplasmic reticulum (ER) stress-induced harmful stimuli and mitochondrial dysfunction were intensified in the absence of HAX1, whereas cardiac-specific HAX1 overexpression improved the contractile function recovery and inhibited cardiomyocyte apoptosis in I/R hearts through robust interaction with heat shock protein-90 (Hsp90) [2, 23, 24]. Particularly, such an interaction constrains the binding of cyclophilin-D to Hsp90 in mitochondria, rendering it susceptible to ubiquitin-proteasomal-induced degradation, which consequently results in the protection against the opening of mitochondrial permeability transition pore (mPTP) and reduction of cell death responses. Regarding ER, HAX1 serves as functional component of phospholamban-sarcoplasmic reticulum calcium ATPase (SERCA2a) regulatory complex in modulating Ca^2+^ cycling and cardiomyocyte contractility under the basal condition [23, 25]. Upon I/R stress, HAX1 can sequester Hsp90 from the inositol-requiring enzyme-1 (IRE-1) to phospholamban/SERCA2a regulatome, thereby inhibiting IRE-1-depent ER stress response, which is featured by the reduction of pro-apoptotic transcription factor C/EBP homologous protein and the decreased caspase-12 activity [23].

Recent studies have revealed a novel protein–protein interaction between HAX1 and Sav1 [30] that can attenuate the HAX1-induced anti-apoptotic response [35]. Interestingly, it has been previously reported that Sav1 can bind to Mst1 (mammalian sterile-like kinase-1), an upstream and core component of the Hippo-Yap signaling pathway that regulates cardiomyocyte survival and heart regeneration [18, 41, 56]. Actually, Mst1 serves as a switch between cardiac cell life and cell death through dual regulation of autophagy and apoptosis [13], and participates in the regulation of cardiomyocyte proliferation through mediation of the subcellular distribution of Yap. In combination with Sav1, Mst1 can phosphorylate Lats1 (large tumor suppressor-1), leading to increased phosphorylation levels of Yap [5, 39]. Upon phosphorylation, Yap is retained in cytosol and subjected to proteasomal degradation induced by ubiquitin, whereas the unphosphorylated counterpart can translocate into the nucleus to serve as a co-activator mediating gene transcription [6]. Indeed, as demonstrated by several studies, cardiomyocyte proliferation was increased, and cardiac regeneration was promoted in damaged hearts when nuclear-localized Yap signal was intensified [31, 34, 57].

In the present study, we report an inhibitory effect of HAX1 on Mst1 kinase activity, impeding the signal transduction of the Hippo pathway and leading to the enhanced nuclear entry of Yap. This response can significantly improve cardiac stem cells’ abilities to proliferate, resist hypoxic injury, and control the expression of VEGF. Importantly, the recovery of heart performance was improved after engraftment of HAX1-overexpressing progenitor cells into the damaged heart tissue, associated with an enhanced angiogenic response.

## Materials and Methods

### Plasmid Construction and Preparation of Lentivirus

HAX1 encoding genes and truncates were amplified by PCR and constructed into a pEGFP-C1 vector (Clontech) to establish the HAX1-GFP-expressing vector. They were then amplified and constructed into a plasmid (pJ3H) that was previously tagged with HA. To generate cell populations expressing HAX1 and/or Mst1, these plasmids were transfected or co-transfected into HEK293TN cells using Lipofectamine® 2000 Transfection Reagent (ThermoFisher Scientific) according to the manufacturer’s instructions.

The lentiviral vector backbone pCDH-CMV-MCS-EF1-CopGFP + Puro (pCDH-GFP) was purchased from System Biosciences (Mountain View, CA). The HAX1 gene was constructed into the restriction enzyme sites of pCDH-GFP, and a non-encoding DNA sequence was constructed to serve as a null control. According to the manufacturer's instructions (System Biosciences), pseudo-viral particles were produced in 293TN cells using a pPACK Lentivector Packaging Kit and concentrated by PEG-it Virus Concentration Solution. The target cells were transduced with pseudoviral stock and selected by puromycin (5 µg/mL, Sigma), as reported previously.

### Preparation of Sca1^+^ Cardiac Stromal Cells

All animals were handled in accordance with the Guide for the Care and Use of Laboratory Animals published by the US National Institutes of Health (NIH Publication No.85–23, revised 1996) and the National Research Council Guide for the Care and Use of Laboratory Animals: 8th Edition published by The National Academies Press, 2011, Washington, DC. All animal experimental protocols were approved by the Institutional Animal Care and Use Committee of the University of Cincinnati. Sca1^+^ CSCs were isolated from mouse hearts according to the protocols developed and modified by us [21, 51]. Briefly, 12-week-old male C57BL6 mice (Harlan) were anesthetized by intraperitoneal injection of ketamine/xylazine (87–100 mg and 13–15 mg/kg, respectively). The adequacy of anesthesia was evaluated by monitoring hind limb reflexes. Hearts were extracted and washed with ice-cold PBS. After removal of the aorta, pulmonary artery, and pericardium, the whole hearts were minced and digested for 20 min at 37ºC with 0.1% type-II collagenase (Invitrogen) and 0.01% DNase I (Worthington Biochemical Corporation). The obtained cells were then passed through a 40 µm filter to remove debris and were subsequently fractionated with 70% Percoll (Fluka) and cultured in a maintenance medium containing serum-free DMEM/F12 (Invitrogen) supplemented with B27 (Invitrogen), 20 ng/ml EGF (Sigma), and 40 ng/ml bFGF (basic fibroblast growth factor, Peprotech). One week later, the cells were transferred to new dishes with a serum-free maintenance medium at a density of 100 cells/cm2. Each colony was mechanically picked up to initiate colony formation in individual sub-cultures in 24 well dishes in DMEM/F12 (Invitrogen) supplemented with 2% FBS, B27 supplement, 20 ng/ml EGF, 40 ng/ml bFGF, and 10 ng/ml LIF (Leukemia inhibitory factor, Millipore). Colony-derived cells were re-seeded on new dishes at 90% confluence and were maintained with DMEM/F12 with 2% FBS. The Sca1 cell-enriched population was also purified from the heart-derived cells with a Magnetic Cell Sorting system anti-Sca1 MicroBead Kit (Miltenyi Biotec) per instructions of the manufacturer and maintained in an expansion medium like other colony-derived cell populations.

### Mst1 Kinase Activity Assessment

Mst1 Kinase activity was assessed using an MST1 Kinase Enzyme System (Promega, Cat. No: V4152). Briefly, Mst1 kinase (in concentration gradient) was incubated with recombinant HAX1 (0.2 μg/μl) (Novus Cat. No: NBP2-23,101) for 1 h at 37℃ and then the kinase reaction was performed according to manufacturer’s instructions. ADP-Glo™ Reagent was then added to stop the kinase reaction and deplete any unconsumed ATP, leaving only ADP. After incubation at room temperature for 40 min, the conversion of ADP to ATP was performed through incubation with Detection Reagent (60 min at room temperature), by which luciferase and luciferin can be introduced to detect ATP. Finally, luminescence was measured with a plate-reading luminometer (Promega), and the EC_50_ was calculated from the corresponding Mst1-dose response curves.

### Co-Immunoprecipitation and Western Blot

HEK293TN cells were grown in 10-cm dishes and transfected with the appropriate plasmids. Cell lysates were incubated with 2 μg of antibody on a rotator overnight at 4 °C. The protein–antibody–protein A-agarose complexes were prepared by adding 50 μl of protein A/G-agarose beads (Santa Cruz) for 1 h at 4 °C. After extensive washing with RIPA lysis buffer, the immunoprecipitated complexes were suspended in reducing sample buffer and boiled for 10 min. After centrifugation to pellet the agarose beads, supernatants were subjected to SDS–polyacrylamide gel electrophoresis (PAGE) and WB.

### Real-Time PCR

Total mRNA was isolated from cell lysates using miRNeasy Mini Kit (Qiagen) according to the manufacturer's instructions. The concentration of RNA was determined spectrophotometrically. Specific real-time PCR primers targeting total XBP-1, spliced XBP-1, and VEGF were used to quantify expression levels. The total XBP-1 primers were: 5’-TCCGCAGCACTCAGACTATGT-3’ as the forward primer and 5’-ATGCCCAAAAGGATATCAGACTC-3’ as the reverse primer. The forward and reverse primers for the spliced XBP-1 were: 5’-GAGTCCGCAGCAGGTG-3’ and 5’-GTGTCAGAGTCCATGGGA-3’, respectively. Glyceraldehyde-3-phosphate dehydrogenase (GAPDH) was used as a reference gene and the primers were: 5'-TCAACAGCAACTCCCACTCTT-3’ as forward primer and 5'-ACCCTGTTGCTGTAGC CGTATTCA-3’ as reverse primer. Real-time PCR was performed on a C1000 Touch Thermal Cycler (Bio-RAD). Relative expression of mRNA was calculated using the comparative threshold cycle (Ct) method.

### Chemotaxis Experiments for Cell Migration

To access migration capacity, CSCs were labeled with DilC12(3) (1,1'-Didodecyl-3,3,3',3'-Tetramethylindocarbocyanine Perchlorate) fluorescent dye (Cat No. 354218 BD Biosciences) through 1-h incubation at 37℃. A cell suspension (300μL) containing 3 × 106 cells/mL in serum-free media was then seeded to an HTS FluoroBiokTM Multiwell (24-well format with 3 μM pore) insert system (Cat No. 351155, BD Falcon). A 500uL of culture media containing SDF1α (100 ng/mL) was added to the lower well of the migration plate, and the cells were incubated under 37℃. The migrated cells were quantified by a Dual-GloTM assay system (Promega) by recording the fluorescence intensity from the plate bottoms at 6 and 12 h post-culture.

### Flow Cytometry

Cells were trypsinized and harvested at 12 h post serum starvation-induced synchronization. Cells were then washed with cold PBS and fixed with 70% ethanol at 4 °C, washed twice with cold PBS, and then subjected to RNase/propidium iodide (PI) staining using Cell Cycle kit (FxCylce™ PI/RNase staining solution, ThermoScientific, F10797) according to the manufacturer's instructions. The cells were analyzed using flow cytometry (BD FACS Canto I), and a minimum of 10,000 events were analyzed at 561 nm excitation wavelength. The data was analyzed using the computational software FlowJo (Version 10. Tree Star Inc. Ashland, OR). The distributions of cell population in phases of G_0_/G_1_, S, and G_2_/M were calculated using the Dean-Jett-Fox mathematical model [22].

For cell death analysis, CSCs were harvested 48-h post hypoxic treatment (20% CO_2_ and 1% O_2_). Briefly, cells were gently washed and stained with PE-Cy7-conjugated Annexin V dye (eBioscience) for 25 min. Stained cells were then washed gently and re-suspended in a solution containing eFluor-780 (eBioscience). Fluorescence signals from PE-Cy7 and eFluor-780 excited at 488 nm and 633 nm were collected at emission wavelengths of 767 nm and 780 nm, respectively, in > 10,000 cells per sample group. Cell distribution diagrams were generated according to fluorescence intensity to calculate the percent of Annexin V positive apoptotic cells in the total population [4].

Surface and intracellular molecular expression of CSCs was analyzed using multicolor flow cytometry. In brief, CSCs were harvested, washed, and suspended in cold PBS containing 3% FBS and 0.02% NaN3. The cells were then incubated with a mixture of rat and mouse IgG (1:1) to reduce nonspecific binding, followed by serial incubations with saturating concentrations of FITC-conjugated, PE-conjugated, and/or PE–Cy7–conjugated mAbs for 1 h at 4 °C. Isotype-matched mAbs were used in control samples. Total fluorescence intensity was analyzed from approximately 10,000 viable CSCs per sample. Histograms of cell distribution were generated according to fluorescence intensity to calculate the median fluorescence intensity (MFI) of fluorochromes to be used as an indication of expression levels of corresponding markers [58].

### Preparation of MI in Mouse Models

The experimental myocardial infarction model was developed in young female C57BL6 mice (12 weeks old; 20–25 g body weight). Animals were anesthetized by intraperitoneal injection of Ketamine/Xylazine (2.5 mg and 0.5 mg respectively). After tracheal intubation with a 22-gauge intravenous catheter (Exelint International), the respiration of mice was controlled artificially by a mechanical ventilator (Harvard Apparatus). The mouse myocardial infarction model was produced as previously described with minor modifications [36]. Briefly, the heart was exposed by left thoracotomy and the left anterior descending (LAD) coronary artery was exposed by gently retracting the left auricle. LAD was subsequently ligated with 6–0 silk under the microscope. Ten minutes after LAD ligation, 20 µl basal DMEM/F12 medium containing 2 × 10^5^ cells (CSCs^Null^ or CSCs^HAX1^) were injected into the infarction border zone of the infarcted heart in three different areas through 29-gauge needles. After cell injection, the chest was sutured with 6–0 silk, and all mice were allowed to recover. Four weeks later, the hearts were excised for immunocytochemistry staining and morphological analysis.

### Echocardiographic Analysis

Transthoracic echocardiography (Vevo® 2100 Imaging System, VisualSonics) was performed with a 15-MHz probe. Echocardiographic data was analyzed in an unbiased manner that was blinded to the experimental groups. Animals were placed supine on an electrical heating pad at 37 °C under light isoflurane anesthesia (usual maintenance level 1.5% isoflurane/98.5% oxygen). Hearts were imaged in 2D long-axis view at the level of the greatest LV diameter with animals under light general anesthesia. This view was used to position the M-mode cursor perpendicular to the LV anterior and posterior walls. LV end-diastolic and end-systolic diameters were measured from M-mode recordings. LV EF was calculated as EF (%) = [LVDd3 − LVDs3/(LVDd)3] × 100. LV minor axis FS was also determined as [(LVDd − LVDs)/LVDd] × 100. All measurements were performed according to the American Society for Echocardiography leading-edge technique standards and averaged over three consecutive cardiac cycles.

### Immunocytochemistry Staining and Morphological Analysis

Hearts were fixed in 4% paraformaldehyde, embedded in paraffin, and then sectioned (4 µm thickness). Tissue sections were rehydrated via sequential treatment with xylene, ethanol (concentration from high to low: 100%, 90%, 80%, 70%), distilled water, and PBS. Trichrome staining was performed as previously described. The LV area images on each slide were filmed using an Olympus BX41 microscope equipped with a CCD (MagnaFire, Olympus) camera. LV fibrosis area and total LV area of each image were measured using Image-Pro Plus software (Media Cybernetics Inc., Carlsbad, CA, USA), and the fibrosis area was calculated as a percentage of the total LV area (fibrosis area/total LV area) × 100%. Antigen retrieval was performed in a microwave (10 min) using citrate buffer (Citric Acid (10 mM), Sodium Citrate (10 mM), pH 7.4). Cultured cells were also fixed using 4% paraformaldehyde and washed before staining. Briefly, samples were blocked for nonspecific staining and then incubated with primary antibodies, and corresponding fluorescent-conjugated secondary antibodies, followed by counterstaining with DAPI (10 μg/mL), and then mounted in VECTASHIELD mounting medium. Immunostaining was observed under the fluorescence microscope (Olympus). Fluorescent imaging was performed with Olympus BX41 microscope and vessel numbers per mm^2^ were counted in 20 randomly selected fields.

### Statistical Analysis

Data were expressed as mean ± SEM. Comparisons were evaluated by Student’s t-test for two groups. Multiple comparisons among three or more groups were performed using one-way ANOVA, and Bonferroni exact test was conducted for post hoc analyses (SPSS 13.0. IBM Co., Armonk, NY). A value of P < 0.05 was considered statistically significant.

## Results

### HAX1 Overexpression Enhances the Proliferation of Sca1^+^ Cardiac Stromal Cells

**Fig. 1:**
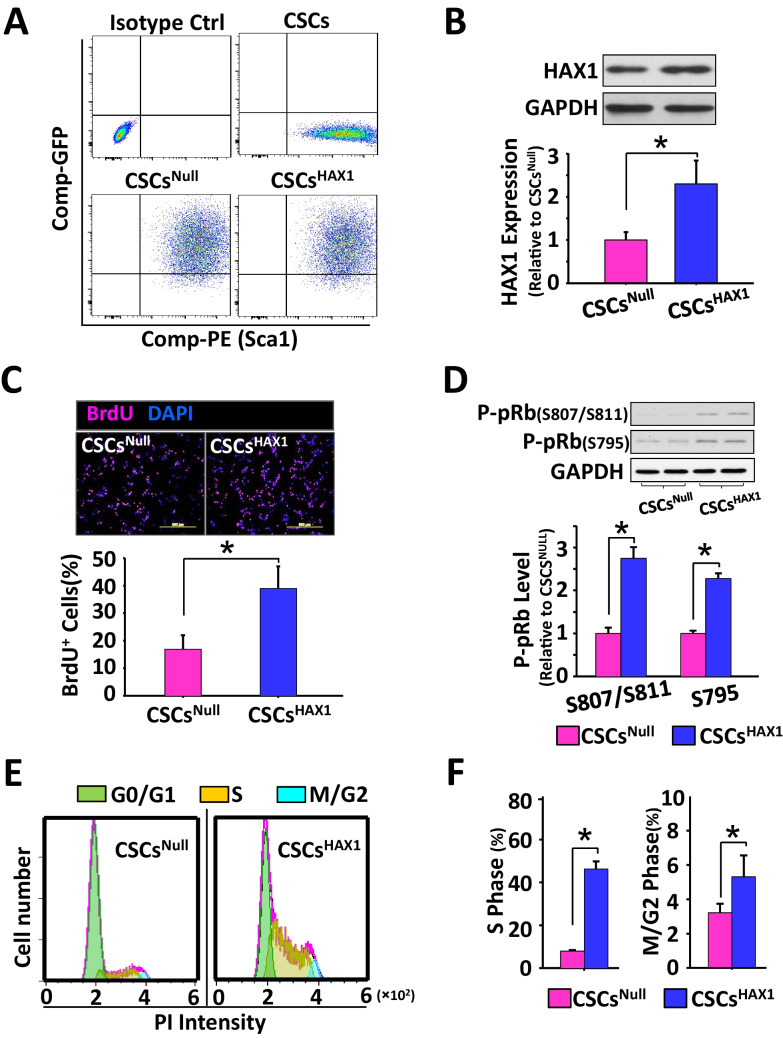
HAX1 enhances the proliferation of cardiac stem cells.** (A)** HAX1-overexpressing CSCs^Sca1+^ and non-encoding gene-expressing Sca1 + cardiac stromal cells (CSCs^Null^), were prepared using lentiviral infection, as indicated by GFP fluorescence. **(B)** Representative Western blots and quantitative data analysis illustrating the expression level of HAX1 in CSCs. Vensus CSC^Null^, * P < 0.05. **(C)** Fluorescence immunostaining was used to indicate incorporation of BrdU into CSCs (Red) and the nuclei were visualized by DAPI staining. Co-localized signals were treated as a proliferative event to be quantified. Vensus CSC^Null^, * P < 0.05. **(D)** Representative Western blots and quantitative data analysis illustrating the expression levels of p-Rb (s807/s811) and p-Rb (s795). Vensus CSC^Null^, * P < 0.05. **(E)** Flow cytometry revealed the distribution of CSCs’ population in G0/G1, S, and M/G2 phases. **(F)** Quantitative analysis of CSCs’ distribution in S phase and M/G2 phase. Vensus CSC.^Null^, * P < 0.05

We examined the impact of HAX1 on the capabilities of stem cells through gain-of-function studies by the use of lentiviruses (Supplemental Fig. 1A). As previously reported [51], mouse Sca1^+^ cardiac stromal cells (CSCs) were successfully isolated and purified as evidenced by over 99% percent Sca1^+^ cell population (Fig. 1A). Fluorescent microscopy (Supplemental Fig. 1B) and flow cytometry analysis (Fig. 1A) showed a pronounced green fluorescence signal indicative of HAX1-overexpressing CSCs (CSCs^HAX1^). Null-gene expressing CSCs (CSCs^Null^) were also successfully generated, and the major progenitor characteristics were not changed in CSCs' response to HAX1 overexpression (Supplemental Fig. 1C). Western blot analysis confirmed a 2.3-fold increase in HAX1 expression in CSCs^HAX1^ when compared with CSCs^Null^ (Fig. 1B). Interestingly, BrdU was more likely to be incorporated into nuclear DNA (increased percentage of BrdU-DAPI co-localization events in CSCs^HAX1^), indicating that HAX1 overexpression can tune up proliferative capacity in CSCs (Fig. 1C). This pro-proliferative effect was supported by analyzing cell-cycle signal cascades. Cyclin D and Cdk4 are prominent upstream components responsible for the phosphorylation modification of retinoblastoma protein (pRb), which is a restrainer of cell-cycle progression from the G1 to the S phase. Upon phosphorylation, this inhibitory effect will be abrogated, and the cell cycle will be promoted. Western blot showed that HAX1 overexpression significantly increased pRb phosphorylation at Ser807, Ser811, and Ser795 (Fig. 1D) but did not affect expression levels of Cyclin D1, Cyclin D3, and Cdk4 (Supplemental Fig. 1D), suggesting potential involvement of alternative cell cycle regulators. Indeed, flow cytometry analysis showed that the percentages of CSCs^HAX1^ population in the S phase and M/G2 phases are 45% and 5% respectively, which significantly outnumbered the corresponding percentages in CSCs^Null^ (10% in S phase and 2.5% in M/G2 phase) (Fig. 1E and F). Collectively, these data provide evidence that proliferative capacity is improved in CSCs response to HAX1 overexpression.

### HAX1 Overexpression Preserves Mitochondrial Function in CSCs to Resist Hypoxic Injury

**Fig. 2:**
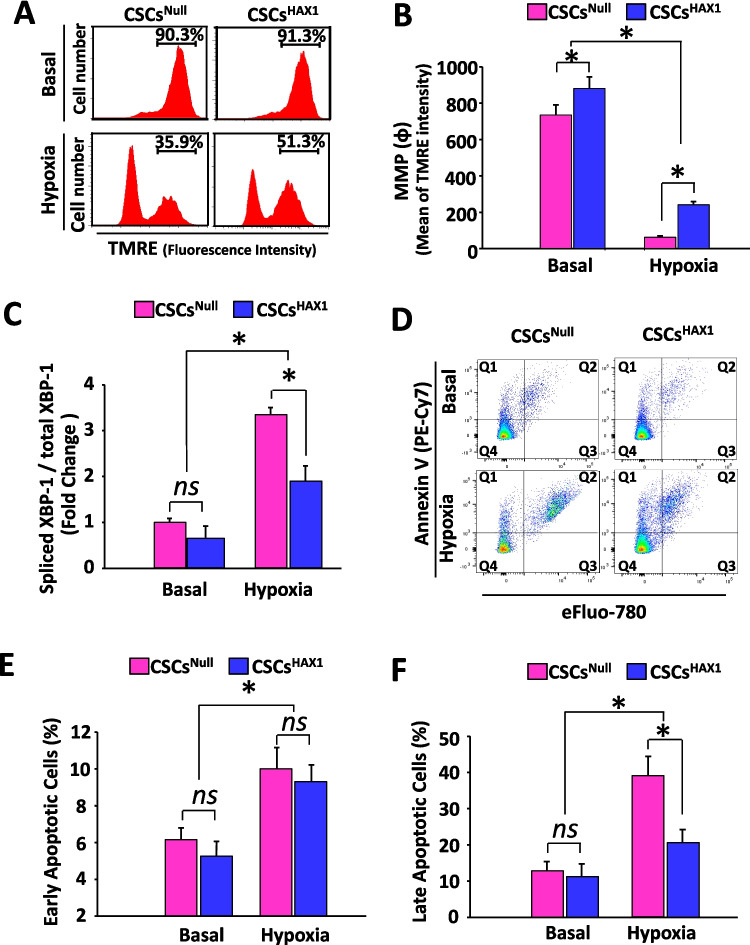
Hypoxia-induced apoptosis is inhibited in CSCs response to HAX1 overexpression.** (A)** Representative histogram of flow cytometry illustrating the distribution of TMRE-loaded CSCs (at 12 h post-hypoxia) according to fluorescence intensity. **(B)** Quantitative analysis of the mean of TMRE intensity illustrating mitochondrial potential. (n = 6 preparations per group; *P < 0.05) **(C)** Quantification of mRNA expression levels of spliced XBP-1 using real-time-PCR. (n = 6 preparations per group; *P < 0.05; ns, no significant difference) **(D)** Representative flow cytometric pseudo-color density plots illustrating the distribution of apoptotic (PE-Cy7 positive) and necrotic (eFluor-780 positive) CSCs. **(E, F)** Quantitative analysis of early apoptotic events **(E)** or necrotic events **(F)** in the absence or presence of hypoxia injury. (n = 6 preparations per group; *P < 0.05; ns, no significant difference)

A recent study demonstrated that cardiac-specific HAX1 overexpression guards against oxidative stress-induced loss of mitochondrial membrane potential (△ψm), resulting in protection against cell death responses in adult cardiomyocytes [24]. Therefore, it would be interesting to investigate whether these protective effects occur in cardiac stem cells. Actually, the CSCs were loaded with a mitochondrial localized fluorescent △ψm indicator (TMRE), enabling △ψm to be monitored before and after exposure to hypoxic conditions. As shown in Fig. 2A, although a similar percentage of cell population with robust MMP was observed in CSCs^Null^ (90.3%) and CSCs^HAX1^ (91.3%) under basal conditions, the quantitative flow cytometric analysis indicated that ψ intensity of CSCs^HAX1^ was higher than that of CSCs^Null^ (Fig. 2B). Under hypoxic conditions, both CSCs^Null^ and CSCs^HAX1^ lost MMP, but 51.3% of CSCs^HAX1^ maintained a robust TMRE signal, compared with 35.9% of CSCs^Null^ (Fig. 2A). Although a reduction of ψ intensity was observed in CSCs^HAX1^ under hypoxic conditions, this value remained at a significantly higher level than that of CSCs^Null^ (Fig. 2B), indicating a protective attribute of HAX1 against mitochondrial membrane potential loss upon hypoxic injury. In agreement with previous studies on adult mouse cardiomyocytes, a reduced ER stress response was also observed in HAX1-overexpressing CSCs. Indeed, the activation of X-box-binding protein-1 (XBP-1) was activated to a greater degree in CSCs upon hypoxia, reflected by its spliced mRNA transcript and assessed by RT-PCR (Fig. 2C). However, there was only a twofold increase detected in the spliced XBP-1 in hypoxic CSCs^HAX1^ compared with basal conditions, which was suppressed at a lower level than that of CSCs^Null^ (threefold).

Alterations in mitochondrial membrane potential and ER stress response are integral to the cell life-death transition, and cell death (including apoptosis and necrosis) is characterized by the exposure of phosphatidylserine at the cell surface and dense clumping of genetic material in the nucleus, which can be indicated by Annexin V-binding and eFluor-labeling, respectively. The early phase of apoptosis can be revealed by Annexin V alone, whereas the late apoptosis appears when cells are double positive in Annexin V and eFluor. As shown in Fig. 2D-F, there was no difference in apoptosis, both in the early (Q1 quadrant) and late phase (Q2 quadrant), between CSCs^Null^ and CSCs^HAX1^ at the basal condition. Although an increased number of apoptotic cells appeared post-hypoxia exposure, a similar degree of early-stage apoptotic response was observed between CSCs^Null^ and CSCs^HAX1^ (Fig. 2D and E).) Interestingly, the percentage of Annexing V/eFluor-double positive cell population was significantly reduced in CSCs^HAX1^ (21%) compared with CSCs^Null^ (39%) (Fig. 2D and F), indicating that overexpression of HAX1 can slow down the late apoptosis response in CSCs post hypoxic challenge. Previous study indicated that the enhanced survival capacity of CSCs is contributed by the increased phosphorylation of AKT [12]. Indeed, the increased AKT phosphorylation was observed in CSCs^HAX1^ compared with CSCs^Null^ (Supplemental Fig. 2).

### Migration Capacity is Increased in HAX1-Overexpressing CSCs

An early report has revealed a significant reduction of CXCR4 in HAX1-deficient B cells [44]. Therefore, it would be interesting to assess CXCR4 expression in CSCs response to HAX1 overexpression. As is shown in Supplemental Fig. 3A and 3B, there was a 1.7-fold increase in CXCR4 expression in CSCsHAX1 compared with CSCsNull. Enhanced CXCR4 expression may promote the migration ability of stem cells through binding with stromal cell-derived factor 1(SDF1) [8]. Indeed, in vitro chemotaxis transwell assay showed that CSCsNull relocated from upper to bottom in the Boyden chamber in the time-dependent pattern after SDF1α exposure (Supplemental Fig. 3C), as evidenced by the increased DilC12(3)’s fluorescence that appeared in the bottom plate (Supplemental Fig. 3D). Particularly, such an SDF1α-induced migration was increased to a greater extent in CSCsHAX1 compared with CSCsNull.

### HAX1 Interacts with Mst1 and Inhibits its Activity

**Fig. 3:**
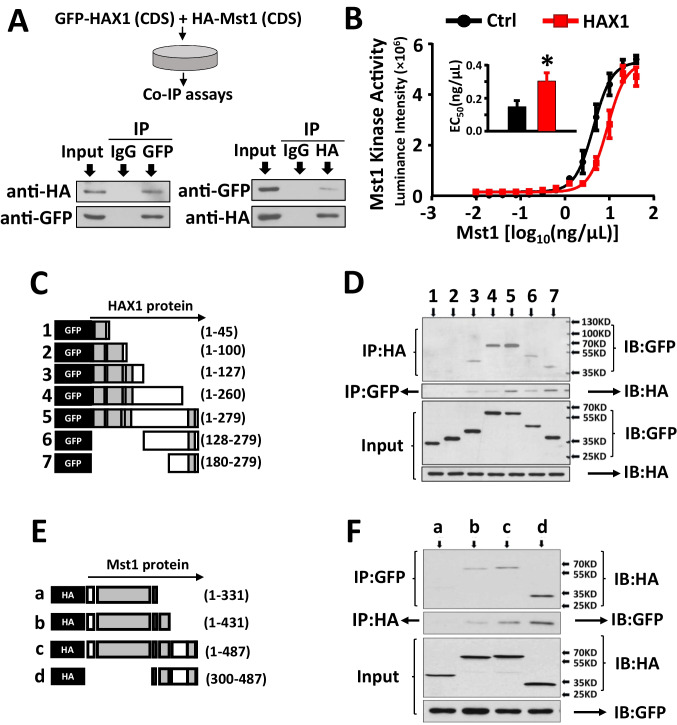
The functional interactions between HAX1 and Mst1** (A)** Co-immunoprecipitation and Western Blot indicate the protein–protein interactions between GFP-tagged HAX1 and HA-tagged Mst1. **(B)** Mst1 enzyme activity was assessed using Ultra-Glo™ luciferase activity assay in the presence of HAX1 or serum albumin (Ctrl), and EC_50_ was calculated according to Mst1 dose–effect curve. **(C)** Truncates of GFP-tagged HAX1-coding sequence were prepared and transfected into HEK293Tn cells with full-length HA-tagged Mst1. **(D)** The binding site of HAX1 to Mst1 was identified using co-immunoprecipitation and Western blot **(E)** Truncates of HA-tagged Mst1-coding sequence were prepared and transfected into HEK293Tn cells with full-length GFP-tagged HAX1. **(F)** The binding site of Mst1 to HAX1 was identified using co-immunoprecipitation and Western blot. (*P < 0.05 *vs*. ctrl)

To establish that a physical interaction exists between HAX1 and Mst1, HAX-1- and Mst1-coding sequences were constructed into GFP- and HA-tagged vectors respectively, which were subsequently co-transfected in HEK293TN cells. Upon pulling down cell lysates with the GFP antibody, a detectable HA signal was observed. Conversely, when cell lysates were pulled down with the HA antibody, a GFP signal became apparent. This reciprocal detection suggests a conclusive interaction between HAX1 and Mst1 (Fig. 3A). This interaction was further confirmed by kinase activity assay on Mst1. Indeed, the half-maximal effective concentration (EC50) of Mst1 kinase (which was calculated from the dose–response curve) was increased from 0.15 (ng/μl) to 0.25 (ng/μl) in the presence of HAX1 (Fig. 3B). To identify the binding site of HAX1 to Mst1, seven GFP-tagged HAX1 cDNA truncates, together with full-length HA-tagged Mst1 gene, were cloned, constructed, and expressed in HEK293TN cells (Fig. 3C). When cell lysates were immune-precipitated using HA antibody and immunoblotted with GFP antibody, a GFP signal appeared in HAX1 gene construct-expressing groups except HAX1 truncates (1–45) and (1–100) (Fig. 3D), indicating that the amino acid residues 100–279 serve as an effective domain of HAX1 binding to Mst1. In the reciprocal experiment, four HA-tagged Mst1 truncates (along with full-length HAX1) were employed to determine the effective binding site of Mst1 to HAX1 (Fig. 3E). Interestingly, the immunoprecipitation and western blot assay revealed this site location at amino acid residues 331–487 at the C-terminal of Mst1 (Fig. 3F).

### HAX1 Regulates the Activation of the Hippo-Yap Complex

**Fig. 4:**
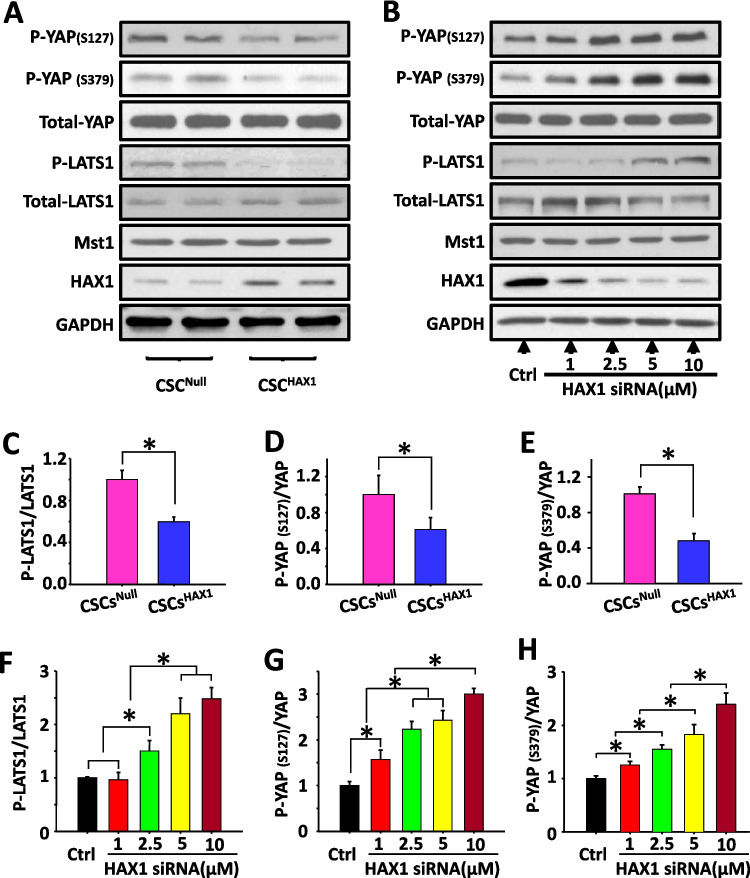
HAX1 is involved in the regulation of Hippo-Yap pathway. **(A, B)** Representative Western blots illustrating the expression level of p-YAP(s127), p-YAP(s379), YAP, p-LATS1, LATS1, Mst1, HAX1, GAPDH in CSC^HAX1^ and CSC^Null^**(A)**, and inCSCs response to HAX1siRNA**(B)**. **(C, D, E)** Quantitative analysis of p-LATS1 **(C)**, p-YAP (s127)** (D)**, p-YAP (s379)** (E)** in CSC^HAX1^ and CSC^Null^. **(F, G, H) **Quantitative analysis of p-LATS1 **(F)**, p-YAP (s127) **(G)**, p-YAP (s379) **(H)** in CSCs response to HAX1 siRNA at gradual concentration level. (n = 6 preparations per group; *P < 0.05)

Mst1 is the core component regulating the Hippo pathway. To assess whether HAX1 overexpression and the associated inhibitory effect on Mst1 resulted in any alterations of Hippo components, quantitative immunoblotting was performed using CSCs. As shown in Fig. 4A, Mst1 expression level was unchanged, but kinase activity was reduced in CSCs^HAX1^, as evidenced by the decreased phosphorylation level of its substrate, large tumor suppressor kinase 1 (LATS1) (Fig. 4C). This inhibition subsequently decreased the phosphorylation of Yap at residues Ser379 and Ser127 (Fig. 4A, D, E). In loss-of-function experiments, endogenous HAX1 gene transcription was silenced using target-specific siRNA, and the dose-dependent reduction in HAX1 expression was indeed observed in siRNA-treated CSCs, which was not associated with alteration in Mst1 expression (Fig. 4B). Correspondingly, the upregulation of LATS1 phosphorylation, associated with enhanced phosphorylation level of Yap, was revealed in CSCs response to HAX1 reduction (Fig. 4B, F, G, H). Thus, the signal transduction of Hippo-Yap pathway in CSCs appears to be determined by the expression level of HAX1.

### HAX1 Overexpression Enhances Nuclear Entry of Yap in CSCs

**Fig. 5:**
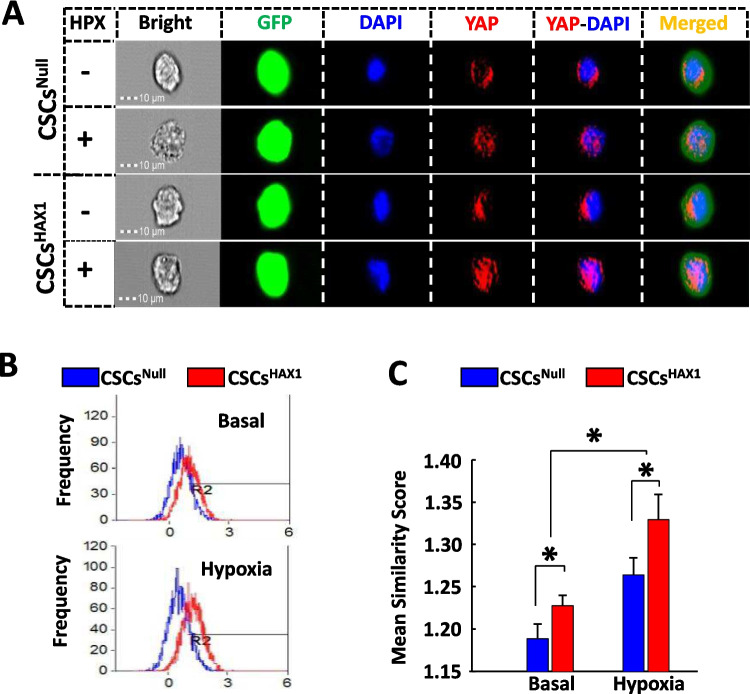
HAX1 overexpression promotes nuclear translocation of Yap in CSCs. **(A)** Nuclear translocation of Yap was investigated in CSCs using ImageStream flow cytometry in the presence and absence of hypoxia (HPX).** (B)** Representative histogram illustrating the distribution of nuclear-located YAP in CSC^NULL^ or CSC^HAX1^ in the absence and presence of hypoxia. **(C)** Quantitative analysis of nuclear-located Yap in CSC^NULL^ or CSC^HAX1^ in the absence and presence of hypoxia. (n = 7 preparations per group; *P < 0.05)

In the absence of modified phosphorylation, Yap can relocate into a cell nucleus and function as a regulator of gene transcription [5]. We therefore assessed whether HAX1-induced inhibitory effect on Yap phosphorylation would promote nuclear entry of this Hippo effector, which can be visualized using image-stream flow cytometry (Fig. 5A). The frequency and intensity of such a nuclear translocation process can be revealed by histogram for frequency distribution of Yap-DAPI colocalization (Fig. 5B) and quantified using “similarity score” ( Fig. 5C ). Some Yap appeared in the nucleus under basal conditions, and Yap-DAPI colocalization was indeed promoted to a greater degree in CSCs^HAX1^ than in CSCs^Null^ (Fig. 5A-C). Interestingly, nuclear entry of Yap was intensified to a higher level under hypoxic conditions, which produced a more pronounced CSC response to HAX1 overexpression (Fig. 5A-C).

### Enhanced Pro-Angiogenesis Effects of HAX1-Overexpressing CSCs

**Fig. 6:**
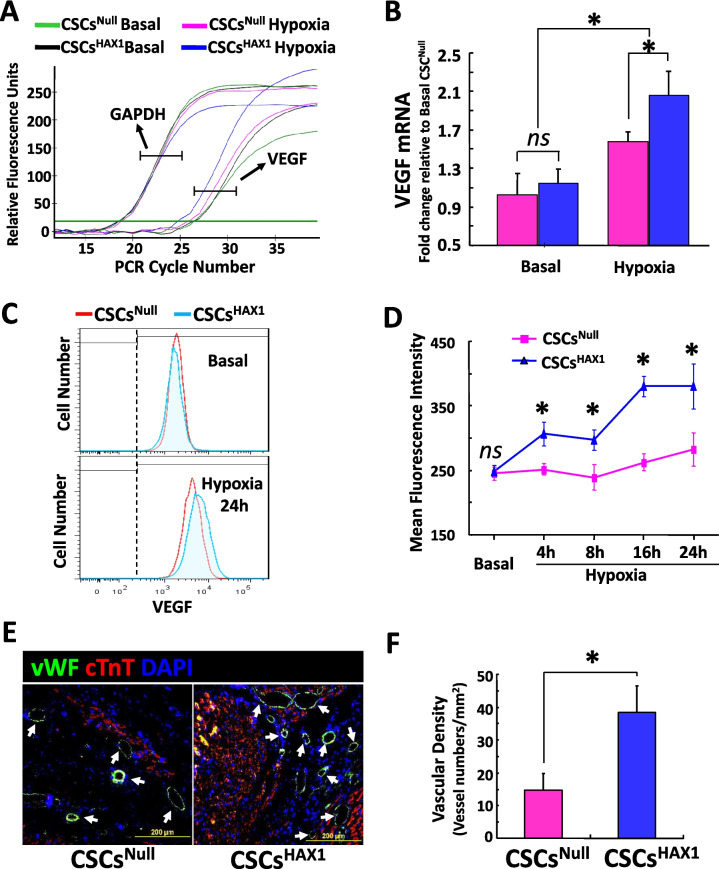
Angiogenic effects are enhanced in HAX1-overexpressing CSCs. **(A) **Representative real-time PCR curve illustrating VEGF and GAPDH mRNA expression in CSC^NULL^ and CSC^HAX1^, in the absence and presence of hypoxia. **(B)** Quantitative analysis of VEGF mRNA expression level relative to basal CSC^NUL^ after normalized to GAPDH levels. (n = 6 preparations per group; *P < 0.05; ns, no significant difference) **(C, D)** VEGF protein expression is assessed using flow cytometry. **(C)** Representative histogram illustrating the distribution of CSCs according to VEGF expression levels at basal conditions and after 24-hour hypoxia exposure. **(D)** Quantitative analysis of VEGF expression levels at basal conditions and 4, 8, 16, and 24 hours post hypoxia exposure. (n = 7 preparations per group; *P < 0.05) **(E)** Representative immunofluorescence staining for von Willebrand factor (vWF) in mouse hearts at 4 weeks post myocardial infarction and CSCs implantation. Nuclei and cardiomyocytes were indicated by DAPI and α-Sarcomeric actin respectively. **(F)** Quantitative analysis of neo-vascular density in infarcted mouse myocardial responses to CSCs implantation. All values were expressed as mean ± SEM. *p < 0.05 was considered statistically significant; n = 6 in each group.

Upon nuclear translocation, Yap can serve as a co-activator of Tead (TEA domain transcription factor) to be involved in gene transcription regulation. Experimental evidence has been documented that Yap promotes angiogenesis by regulating the expression of secreted proangiogenic proteins [38], whereas pharmacological inhibition of Yap-Tead signaling downregulated the expression level of vascular endothelial growth factor VEGF [3, 33, 43]. Considering these previous studies, we assessed the VEGF expression and pro-angiogenetic effects in HAX1-overexpressing CSCs. Under basal conditions, there was no significant difference in the gene expression level of VEGF between CSCs^Null^ and CSCs^HAX1^. After exposure to hypoxia, the gene transcription level of this potent angiogenic factor was increased in both groups, but the enhancement in HAX1-overexpressing CSCs was significantly higher compared with CSCs^Null^ (Fig. 6A and B). Such a pro-angiogenic effect was further confirmed on protein expression level through performing flow cytometry assessment on CSCs at multiple time points. As is shown in Fig. 6C and D, the VEGF expression level was similar between the two groups prior to hypoxia. However, after hypoxic exposure, HAX1-induced pro-angiogenesis was revealed by the pronounced enhancement of VEGF expression in CSCs^HAX1^ compared with CSCs^Null^, which appeared at 4 h and peaked at 16 h post hypoxia (Fig. 6C and D). To investigate whether HAX1-induced pro-angiogenesis can be maintained in ischemic heart tissue, myocardial infarction was simulated via performing permanent LAD ligation on mouse heart and CSCs were subsequently engrafted into the injured myocardium. Indeed, ischemic injury resulted in the loss of cardiomyocytes as evidenced by the disappearance of cTnT signals in the damaged site (Fig. 6E). The neovascularization response can be indicated by the appearance of vWF expression in the infarcted myocardium, which was actually observed post the implantation of CSCs (Fig. 6E). Remarkably, the vascular density was increased to a greater degree in CSCs^HAX1^-engrafted myocardial tissue than that of CSCs^Null^ group (Fig. 6F), indicating that HAX1 overexpression can promote the angiogenic effects of CSCs both in vitro and in vivo.

### Enhancement of Contractile Function in Ischemic Heart Post-Implantation of HAX1-Overexpressing CSCs

**Fig. 7:**
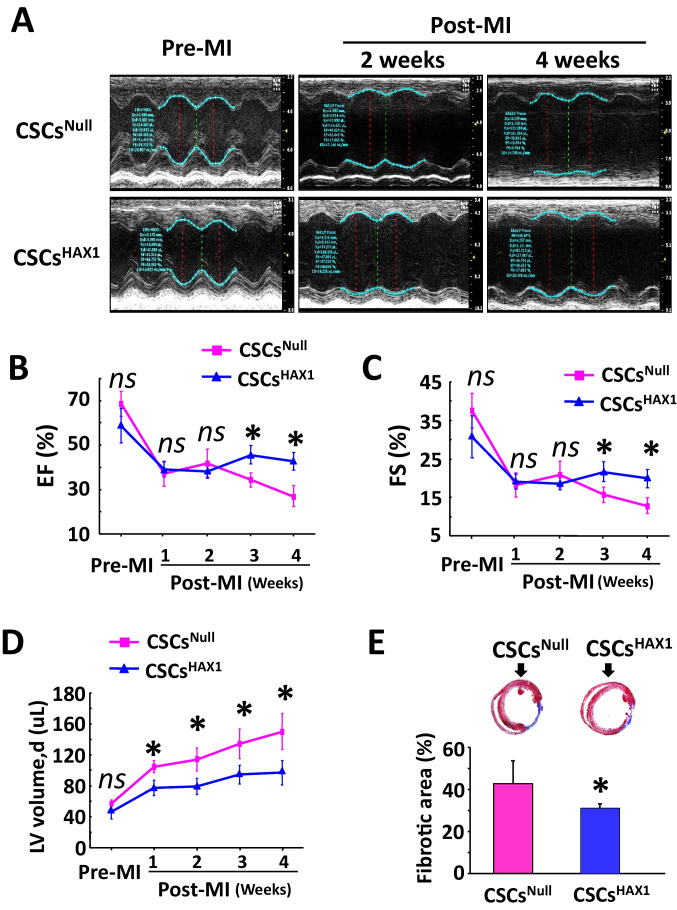
Implantation of CSC^HAX1^ significantly improved cardiac function recovery post myocardial infarction. **(A)** Representative M-mode echocardiography illustrating cardiac function under basal conditions and at 2 and 4 weeks post MI. **(B, C, D)** Quantitative analysis of ejection fraction (EF) **(B)**, fraction shortening (FS) **(C)**, and left ventricular diastolic volume (LVd) **(D)** under basal conditions and at 1, 2, 3, and 4 weeks post MI. **(E)** Representative heart sections after Masson's trichrome staining and quantitative analysis of fibrotic area (blue). Vensus CSC^Null^, * P < 0.05, ^*ns*^ no significant difference

Heart performance and cardiac remodeling after MI injury in CSC-implanted hearts were investigated as well. The CSCs were suspended in PBS and then injected into mouse heart tissue, therefore, PBS-treated MI mice were served as control. The cardiac function was compromised in PBS-treated mice and such heart failure appeared from the 1 week post-MI, as evidenced by the decreased EF% and FS%. Importantly, both EF% and FS% reduced at 1 week and decreased to 15% and 8% respectively at 4 weeks post-MI (Supplemental Figs. 4A-4C). CSC implantation-induced improvement in cardiac function emerged in the third-week post-MI. Actually, the percentages of EF and FS were enhanced to 30% and 18% respectively in CSCs^Null^-treated hearts, and such an improvement was increased to a greater extent in CSCs^HAX1^-implanted hearts, which still remained at 4 weeks post-MI (Fig. 7A, B, C). Along with the beneficial function improvements, the echocardiographic analysis indicated that HAX1-overexpressing CSCs also alleviate MI-induced cardiac remodeling. Indeed, from 2 weeks post MI, the left ventricular diastolic volume continuously remained at a lower level in CSCs^HAX1^-engrafted hearts than CSCs^Null^-engrafted group (Fig. 7D), and histological analysis of these hearts 4 weeks after MI showed a reduction of fibrotic area in CSCs^HAX^ group compared with CSCs^Null^ treatment group (Fig. 7E).

## Discussion

This study provides novel experimental evidence that HAX1 can interact and inhibit the kinase activity of Mst1, modulating the Hippo-Yap pathway and regulating cell survival under hypoxic conditions. Silencing endogenous HAX1 expression increased the transduction of Hippo signals, whereas overexpression of HAX1 diminished the function of Hippo complex and led to the enhanced nuclear entry of Yap in cardiac stem cells, associated with an increased ability to proliferate, enhanced ability to survive against hypoxia, and promoted competency to stimulate angiogenesis. Significantly, these advantageous responses in CSCs amplify their therapeutic impact on myocardial infarction. This is evidenced by improved recovery in cardiac function, a reduction in fibrotic areas, and enhanced neovascularization within the infarcted myocardium.

Several proteins can interact with HAX1 to modify the mitochondrial-dependent apoptotic pathway [45]. Preliminary reports have indicated that HtrA2 (High-temperature requirement protein A2) can proteolytically cleave HAX1 upon the induction of apoptosis [9], and the subsequent study indicated that such a process actually serves as an aspect of HAX1-induced protective mechanisms in mitochondria [1, 14]. Indeed, HAX1 can present HtrA2 to Parl (presenilin-associated, rhomboid-like), a protease localized in the mitochondrial intermembrane space [7, 19], thereby blocking the release of HtrA2 from mitochondria into cytosol and protecting XIAP (X-linked inhibitor of apoptosis) from degradation [20], consequently attenuating mitochondria-induced apoptosis. A previous study has demonstrated that the binding of HAX1 to the N-terminal fragment of the Hsp90 can alleviate ER stress response to promote survival in cardiomyocytes upon the ischemia–reperfusion injury [23], and the most recent study revealed the contribution of this affinity binding to the maintenance of mitochondrial membrane function in cardiomyocytes [24]. Particularly, HAX1-mediated interference of cyclophilin-D binding to Hsp90 in mitochondria renders it susceptible to ubiquitin–proteasome-dependent proteolysis, thereby resulting in protection against cyclophilin-D-dependent mPTP activation [24, 48].

Our present findings suggest another protective mechanism of HAX1, functioning through interaction and inhibition of Mst1 kinase activity. Particularly, the residues 100–279 at the C’-terminus of HAX1 appeared as an important motif for targeting Mst1, which also serves as effective domains of this multiple functional protein for the interactions with HtrA2 and caspase-9 [15]. It has been accepted that Mst1 acts as a response kinase to phosphorylate Beclin1 at threonine-108 upon stress stimuli, leading to the separation of Beclin-1 from the Atg14L-Vps34 complex. The dissociated Beclin1 subsequently competes with Bax to form complexes with Bcl-2, which can suppress autophagy and result in the accumulation of protein aggregates [37]. Alternatively, Mst1 can phosphorylate BCl-XL at Serine in the BH4 domain, thereby obstructing the Bcl-xL/Bax covalent bindings and subsequently initiating an apoptotic response post-translocation of Bax to mitochondria [11]. Initiation of an inhibitory phosphorylation mechanism plays a principal role in regulating Mst1 kinase activity. Indeed, enhanced phosphorylation levels at Threonine-183 and Tyrosine-433 will lead to Mst1 activation [49, 55], whereas Mst1 activity will be inhibited when it is phosphorylated on Threonine-120, Theronine-387, and Serine-438 [10, 49]. Although phosphorylation levels of these sites were not profiled in this study, the activity of Mst1 was indeed constrained in the presence of HAX1, as evidenced by the increased EC_50_ value from the curve for enzyme dynamics. Evidence of this inhibition was further supported by the decreased phosphorylation levels of LAST1, a signaling molecule that acts as the substrate of Mst1. Importantly, the C-terminal of Mst1 (residues 331–487) was revealed as the target motif of HAX1 containing two crucial regulatory domains: an auto-inhibitory domain (residues 331–394) that modulates kinase activity and a SARAH (Sav/Rassf/Hippo) domain (residues 431–487) that regulates the dimerization of this kinase [47]. HAX1-induced functional inhibition of Mst1 not only protected mitochondria against oxidative insult and consequently kept them from hypoxia-induced cell death, but also participated in the proliferation mechanism and the genomic regulatory programming in cardiac stem cells.

Increased HAX1 expression has been revealed in several cell lines with pronounced proliferation capacity [16, 28, 29, 54], and was also observed in the current study when HAX1 was exogenously overexpressed in CSCs, as evidenced by the increased phosphorylation level of pRb and the enhanced distribution of cell population in DNA synthetic stage. Such an effect might be attributed to HAX1-overexpression-induced nuclear entry of YAP and subsequent coordination with TEAD1, which is a Hippo signaling effector and an integral module of the transcription factor combinatorial control of cell proliferation [38]. In addition, YAP activation together with TEAD can directly activate *Pi3kcb* expression via covalently binding to an enhancer in the first intron of *Pi3kcb*, which consequently promotes cardiomyocyte proliferation and heart regeneration via an AKT-dependent mechanism [32]. Previous studies showed that YAP/TEAD activation promotes tube formation of human microvascular endothelial cells [38], whereas pharmacological inhibition of YAP-TEAD signaling significantly reduced the expression of VEGF [3], an important signaling protein involved in both vasculogenesis and angiogenesis. In the present study, increased VEGF expression was observed in HAX1-overexpressing CSCs at both gene transcription and protein levels, suggesting that HAX1 may facilitate YAP-TEAD to target the enhancer of angiogenic genes. Importantly, HAX1-induced pro-angiogenic paracrine effects in Sca1^+^ CSCs led to a pronounced tissue repair process in the injured myocardium, as evidenced by the increased formation of new blood vessels in the infarcted region. Such a beneficial response significantly improves the therapeutic efficacy of CSCs in ischemic hearts. Indeed, although engraftment of Sca1^+^ CSCs can improve the recovery of heart contractile function, cardiac remodeling was not attenuated in infarcted hearts.

Our recent study demonstrated that post-transplantation of HAX1-overexpressing Sca1^+^ CSCs improved MI-induced fibrosis formation and left ventricular remodeling in injured hearts. Myocardial remodeling following cardiomyocyte loss is a multifaceted process involving various cell components, including fibroblasts, endothelial cells, and immune cells. The Hippo-Yap pathway regulates cell–cell communications among tissue-resident cells and infiltrating immune cells through direct interactions, soluble factors or cytokines, extracellular vesicles, and the extracellular matrix, thereby controlling biological and pathological processes [42, 59]. For instance, Hippo-Yap signaling in epicardial cells has been found to play a critical role in post-MI recovery by mediating interferon-γ expression and recruiting T-regulatory cell infiltration [46]. Notably, Hippo-Yap activation in cardiac fibroblasts post-MI can induce a pro-fibrotic response and contribute to proinflammatory macrophage activation or migration by regulating interleukin-33 expression [40]. Therefore, it is important to note that Hippo signaling may exert various effects depending on different cell contexts in the cardiovascular system and microenvironments [53]. In future studies, we will continue to explore the role of the HAX1-Hippo signaling axis in potential cell–cell communications between CSCs and immune cell infiltration, thereby contributing to the improvement of myocardial remodeling post-MI.

In summary, this is the first study to reveal the interactive relationship between HAX1 and Mst1, through which the Hippo-Yap signal activation can be modulated. The identification of the effective interaction fragments between these two macromolecules allows the regulatory mechanism of Mst1 kinase to be further understood and provides valuable preliminary data to develop potential therapeutic agents targeting Mst1. HAX1-induced functional inhibitory effect on Mst1 confers resistance to the dissipation of mitochondrial membrane potential and protects against hypoxia-induced cell death. HAX1 overexpression-induced reduction of Hippo activity and enhanced nuclear entry of Yap promotes the VEGF expression of CSCs. Such beneficial effects promoted the angiogenic response in CSCs-engrafted hearts post-myocardial infarction. Hence, HAX1 is an important signal node to consider for cardiac stem cell intervention in the treatment of ischemic heart disease.

### Supplementary Information

Below is the link to the electronic supplementary material.

Supplementary file1 (PDF 808 KB)

## Data Availability

All data from this study are available from the article and supplementary files, and other resources used in this study are available from the corresponding author upon reasonable request.
