# Supplementary material for: HAX1-Overexpression Augments Cardioprotective Efficacy of Stem Cell-Based Therapy Through Mediating Hippo-Yap Signaling
Source: Stem Cell Rev Rep. 2024 May 7;20(6):1569–86. doi: 10.1007/s12015-024-10729-z (PMC11319392; doi:10.1007/s12015-024-10729-z)
Supplement: Supplementary file 1 — Supplementary file1 (PDF 808 KB) [file 12015_2024_10729_MOESM1_ESM.pdf]

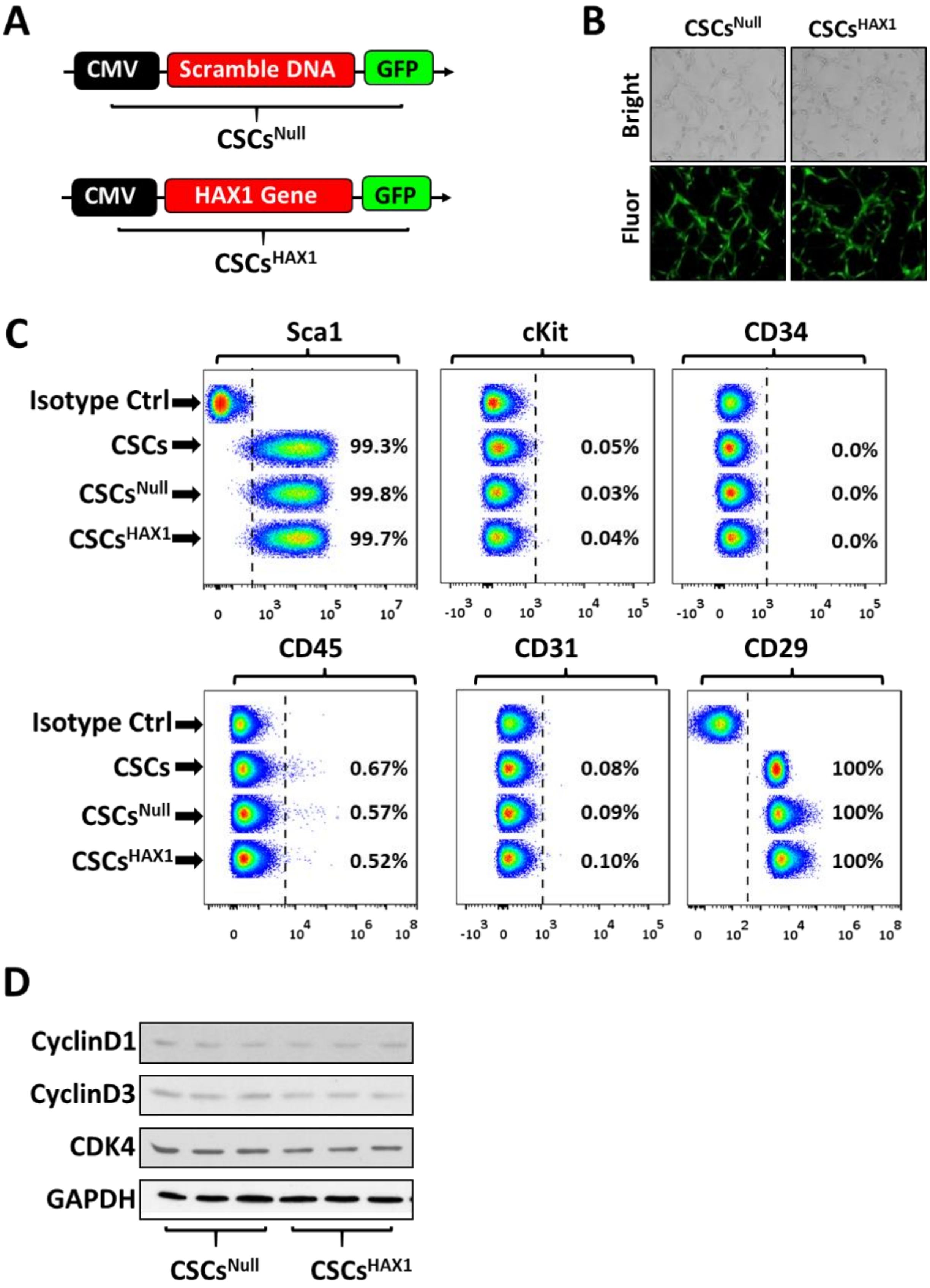


**Supplemental Figure 1. Preparation and characteristics of HAX1-overexpressing Sca1$^{+}$ cardiac cells.** (**A**) Schematic diagram of the recombinant lentiviral vector. Mouse HAX1-encoding gene as well as a scramble DNA sequence were amplified by PCR and incorporated into the downstream of CMV promoter in the lentiviral backbone vector, which were subsequently transduced into Sca1$^{+}$ cardiac stromal cells (CSCs) to build up CSCs$^{HAX1}$ and CSCs$^{Null}$ cell lines. (**B**) CSCs appeared infected by nearly 99% after lentiviral gene transfer, as indicated by the appearance of GFP fluorescence. No morphological changes were detected in CSCs$^{HAX1}$ and CSCs$^{Null}$. (**C**) Characteristics of CSCs, CSCs$^{Null}$, and CSCs$^{HAX1}$ as indicated by Sca1, cKit, CD31, CD34, CD45, and CD29, were assessed by flow cytometer. (**D**) Representative Western blots illustrating the expressions of CyclinD1, CyclinD3, CDK4 and GAPDH in CSCs$^{HAX1}$ and CSCs$^{Null}$.

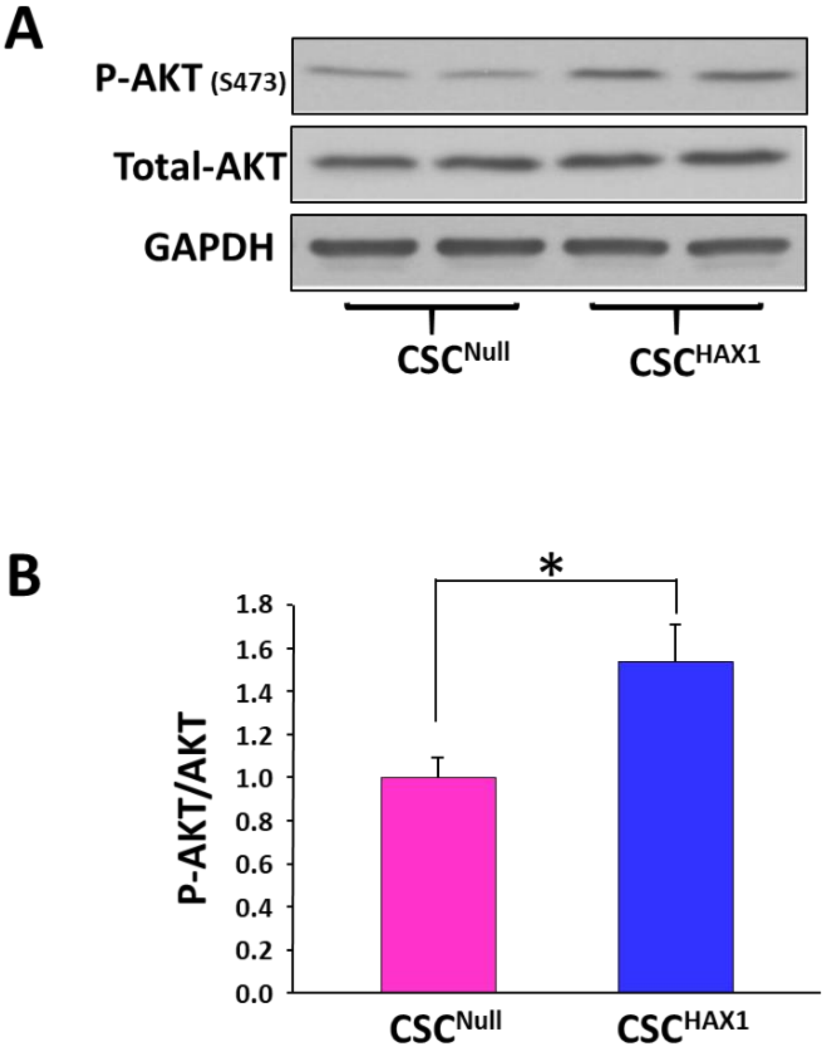


**Supplemental Figure 2. Phosphorylation of AKT in CSCs response to HAX1 overexpression. (A)** Representative Western blots illustrating the expression level of p-AKT, total AKT, and GAPDH in $CSC^{Null}$ and $CSC^{HAX1}$ **(B)** Quantitative analysis of AKT phosphorylation in $CSC^{Null}$ and $CSC^{HAX1}$(n=6 preparations per group; *P<0.05)

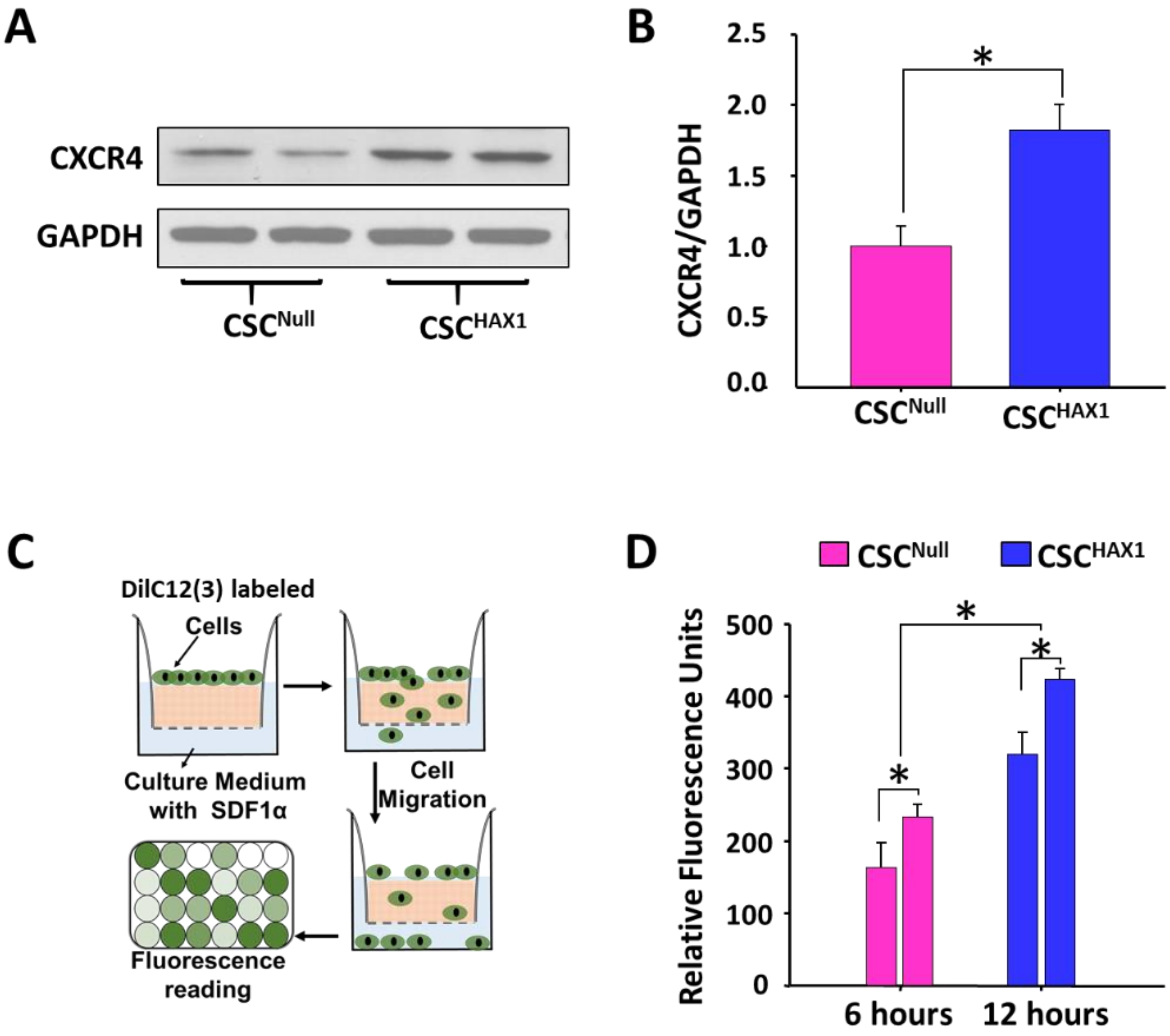


**Supplemental Figure 3. Migration capability is enhanced in CSCs response to HAX1 overexpression. (A)** Representative Western blots illustrating the expression level of CXCR4 and GAPDH in $CSC^{Null}$ and $CSC^{HAX1}$. **(B)** Quantitative analysis of CXCR4 expression in $CSC^{Null}$ and $CSC^{HAX1}$. **(C)** Diagram of chemotaxis experiment to evaluate migration ability in CSCs. **(D)** Quantitative assessment of CSCs migration ability through recording fluorescent intensity in the bottom chamber. (n=6 preparations per group; *P<0.05)

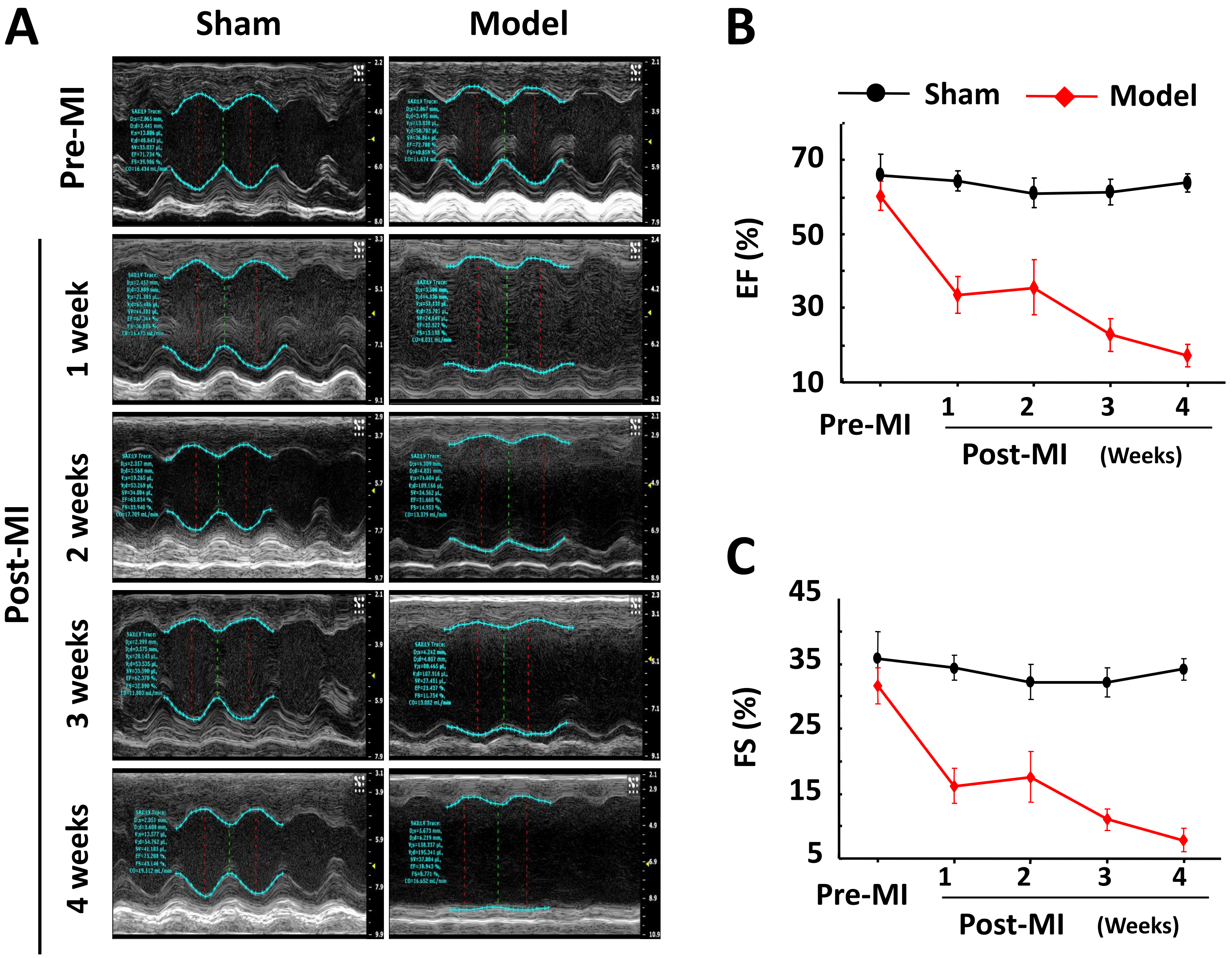


**Supplemental Figure 4. Contractile function compromised in mouse hearts post myocardial ischemic injury. (A)** Representative M-mode echocardiography illustrating cardiac function under basal conditions and at 1, 2, 3 and 4-week post MI. (**B, C**) Quantitative analysis of ejection fraction (EF) **(B)**, fraction shortening (FS) under basal conditions and at 1, 2, 3, and 4 weeks post MI. All values were expressed as mean±SEM. $*p<0.05$ was considered statistically significant; n=6 in each group
